# Diet Quality, Saturated Fat and Metabolic Syndrome

**DOI:** 10.3390/nu12113232

**Published:** 2020-10-22

**Authors:** Stéphanie Harrison, Patrick Couture, Benoît Lamarche

**Affiliations:** 1 Centre Nutrition, santé et société (NUTRISS), Institut sur la nutrition et les aliments fonctionnels (INAF), Université Laval, Québec, QC G1V 0A6, Canada; stephanie.harrison.1@ulaval.ca (S.H.); patrick.couture@fmed.ulaval.ca (P.C.); 2 School of Nutrition, Université Laval, Québec, QC G1V 0A6, Canada; 3 CHU Research Center, Québec, QC G1V 0A6, Canada

**Keywords:** saturated fatty acids, metabolic syndrome, diet quality, dietary guidelines, cardiovascular disease

## Abstract

Indices reflecting overall diet quality are used globally in research to predict the risk of various diseases and metabolic disorders such as metabolic syndrome (MetS). Such indices are built to measure adherence to current dietary guidelines or to best assess the diet–disease relationship. Although mostly food-based, dietary guidelines often include recommendations to limit saturated fatty acid (SFA) intake in order to prevent cardiovascular diseases. However, not all diet quality indices consider SFA in their definition of diet quality. Additionally, the relationship between SFA consumption and the development of MetS remains unclear. The purpose of this short review was to explore the association between MetS and various diet quality indices and dietary patterns, with a focus on how SFA contributes to these associations.

## 1. Introduction

Various scores and indices are available to assess overall diet quality in population-based or interventional studies. These scores measure either adherence to certain dietary patterns, such as the Mediterranean diet (MedDiet) or the Dietary Approach to Stop Hypertension (DASH), or to country-specific dietary guidelines, such as Healthy Eating Indices (HEI). As discussed below, dietary patterns and diet quality indices have been associated with the risk of various diseases, including the metabolic syndrome (MetS), a collection of metabolic disorders that increase the risk of cardiovascular diseases (CVD), stroke or type 2 diabetes [1]. The typical features of MetS are central obesity, insulin resistance, dyslipidemia, hypertension, dysglycemia, and a pro-inflammatory/pro-thrombotic state [1]. The dyslipidemic features of the MetS are hypertriglyceridemia (fasting and postprandial) and low high-density lipoprotein cholesterol (HDL-C). While an elevated low-density lipoprotein cholesterol concentration (LDL-C) is not considered a typical feature of MetS, other features of LDL are. Specifically, patients with MetS generally have smaller and denser LDL particles, which are more prone to oxidative stress and are cleared less rapidly from the circulation than larger LDL particles [2].

Dietary patterns are a multidimensional representation of eating and, by definition, do not focus on singled out nutrients or foods. This is also the case for dietary guidelines, which have shifted from mostly nutrient-based to mostly food-based recommendations in recent years [3]. Nevertheless, most dietary guidelines around the globe still include a recommendation to limit intakes of specific nutrients of public health concern, including saturated fatty acids (SFAs). The recommendation to limit the consumption of SFAs is based largely on their well-established cholesterol-raising effects [4,5]. Data from a number of randomized controlled trials (RCTs) have shown that replacing dietary SFA with unsaturated fats reduces the risk of combined CVD events and are also at the basis of the recommendation to limit SFA intakes [5,6]. Replacing dietary SFA with carbohydrates from whole grains foods may also yield cardiovascular benefits, unlike carbohydrates from refined grains [7,8,9].

Although SFAs have been considered a major nutritional risk factor of CVD for more than 50 years, recent data have provided new challenging evidence suggesting that the SFA–CVD relationship may not be as straightforward as originally thought [10]. For example, the meta-analysis that showed a significant impact of SFA reduction on combined CVD events and showed no significant effect of SFAs on the risk of fatal myocardial infarction (MI) or coronary heart disease (CHD) mortality [5]. The authors of this meta-analysis emphasized the lack of robust, high-quality data to reach definitive conclusions on many of these associations. Others suggest that the recommendations on healthy dietary patterns capture the inherent risk attributed to variations in SFA intake, implying that specific recommendations on SFA intake are, in that context, redundant [11]. The fact that the increase in LDL-C concentrations seen with higher intakes of SFA intake is generally paralleled by an increase in the size of the LDL is another factor complexifying the association between dietary SFA and CVD risk [11]. Finally, the heterogeneity in the health effects of individual types of SFAs (e.g., myristic, lauric, etc.) [7,12] and the important interindividual variation in response to SFA reduction [13] are additional considerations put forward by those against the specific recommendations on SFA [11].

Beyond its well-known LDL-raising effects, the impact of SFA consumption on cardiometabolic health remains controversial. While data from a recent systematic review suggest that increased intake of SFA is associated with an increased risk of MetS, this association appeared to be dependent on the concurrent variations of other nutrients [9]. The purpose of this short review was therefore to explore the association between MetS and various diet quality indices and dietary patterns, with a focus on how SFA may contribute to these associations.

## 2. Diet Quality and Metabolic Syndrome

### 2.1. The HEI and MetS

The first HEI was developed in 1995 by the United States Department of Agriculture (USDA) in order to measure adherence to the Dietary Guidelines for Americans (DGA) as well as the Food Guide Pyramid. The HEI’s main goal was to serve as a “report card” of Americans’ diet, i.e., a measure of overall diet quality. This first HEI had 10 components related to recommendations found in the Food Guide Pyramid (Grains, Vegetables, Fruits, Milk, Meat, and “Other Foods”) and in the DGA (Total fat, SFA, Cholesterol, Sodium, and Variety). The HEI-2015 is the most recent healthy eating index in the USA and reflects adherence to the 2015–2020 DGA. It has 13 components, including both adequacy and moderation components (Table 1). The moderation components of the HEI-2015 include a fatty acids sub-score calculated as the ratio of unsaturated fat to SFAs, as well as an SFA sub-score. According to the 2015–2020 DGA, SFA intake should not exceed 10%E per day (the SFA sub-score of the HEI-2015) and should be replaced by unsaturated fats (the fatty acids sub-score of the HEI-2015). As indicated above, those recommendations are based on many studies having shown that replacing SFA with unsaturated fat (notably PUFA) reduces serum levels of total and LDL-cholesterol [4], as well as the risk of CVD events [5]. The rationale for the SFA recommendation (<10% E) in the 2015–2020 DGA is not based on the upper limit instated by the Institute of Medicine. It is based on the notion that in most instances, people with an SFA intake > 10% E cannot meet the recommended intake of all food groups while maintaining an energy balance [3]. SFA intake in the calculation of the HEI-2015 is also partially accounted for in the total protein foods adequacy component, higher fat protein foods receiving fewer points than lower fat protein foods [14]. Finally, the adequacy dairy component of the HEI-2015, which rewards a higher consumption of dairy products irrespective of fat content, as well as the SFA moderation component, which sanctions higher SFA intakes, reflects the 2015–2020 DGA recommendations to favor lower-fat or fat-free dairy products [3].

Surprisingly, very few studies have documented the association between HEIs (2010 or 2015 versions) and the risk of MetS. In a cross-sectional analysis of 1036 Iranian women, participants in the highest quartile of HEI-2010 had a 28% lower risk of MetS compared with those in the first quartile (95% CI 0.50–0.96). Abdominal obesity, high blood pressure, high serum triacylglycerol and low serum HDL-C also decreased across HEI-2010 quartiles [15]. Structural equation modeling analyses of data from a sample of 188 healthy obese adults revealed that the HEI-2015 mediated the association between age and several cardio-metabolic risk factors associated with MetS, including fat mass, fat free mass, systolic blood pressure (SBP) and HDL-C. HEI-2015 scores also mediated the association between gender and waist circumference, SBP, triglyceride and HDL-C [16].

The extent to which each of the HEI-2015 sub-scores, including those related to SFA intake, contribute to modifying individual features of the MetS is a complex question. To the extent that high LDL-C concentrations are not a typical feature of the MetS, adequacy or moderation components of the HEI-2015 having an impact on LDL-C concentrations would not predict changes in the incidence of MetS. However, replacing SFA with PUFA or MUFA slightly reduces HDL-C, total cholesterol, and TG concentrations in healthy adults [4]. Furthermore, replacing SFA by PUFA from different oils or foods reduced HDL-C concentrations in a recently published cross-over randomized controlled trial (RCT) of 36 men and women at risk of CVD, while having no effect on serum TG and glucose levels [17]. The substitution of SFA by PUFA, particularly PUFAs from walnuts, had a small but significant lowering effect on blood pressure but no effect on arterial stiffness in the same RCT [17]. These results suggest that dietary SFA, because of opposing effects on many of the typical features of MetS, cannot explain in and of themselves the favorable association between diet quality, as measured by the HEI-2015, and MetS. On the other hand, consumption of other foods and nutrients accounted for in the HEI-2015 has been more consistently associated with specific features of the MetS. For example, high vs. low dairy consumption has been associated with a lower risk of abdominal obesity and of being overweight [18]. The blood pressure lowering effect of a low sodium diet is well established [19]. Data from observational studies indicate that consumption of fruits and vegetables is associated with a lower risk of MetS [20]. Fruit consumption, independent of vegetable consumption, has also been inversely associated with the incidence of hypertriglyceridemia [21]. Higher consumption of sugar-sweetened beverages (SSBs), one of the main sources of added sugar in the North American diet, has been associated with an increased risk of MetS [22]. Taken together, these results suggest that the SFA component of the HEI-2015 per se cannot explain the favorable association between the HEI-2015 and the risk of MetS.

### 2.2. The aHEI and MetS

Unlike the HEI-2015, which is meant to measure adherence to dietary guidelines, the Alternate HEI (aHEI) was developed not only as a measure of diet quality but also to entail the diet–disease association. It was first developed in 2002 and included 9 components, including vegetables, fruits, and cereal fiber [23]. While the first version of the aHEI included an SFA component, the updated 2010 version of the aHEI does not (Table 1) [24], reflecting to some extent the inconsistent association between SFA consumption and CVD [11]. This is not to say that SFA is not indirectly accounted for in the aHEI. Indeed, a high score resulting from a high consumption of vegetables and fruits, whole grains, nuts and legumes, and PUFA, combined with a low consumption of SSBs, sodium, and red/processed meat, reflects a healthy food pattern that is very likely to be low in SFA.

In a cross-sectional analysis of 12,406 US Hispanics and Latinos from the multicenter, population-based Hispanic Community Health Study/Study of Latinos cohort, a higher aHEI was associated with lower odds of MetS [25]. Interestingly, the association of the aHEI and cardiometabolic factors varied by ethnic background. Specifically, the aHEI was inversely associated with waist circumference, blood pressure, and glucose among Mexicans and Puerto Ricans and with TG among Mexicans only, and was positively associated with HDL-C among Puerto Ricans and Central Americans [25]. We have also shown a similar inverse association between the aHEI and the prevalence of MetS in a cross-sectional analysis of 998 men and women from the province of Québec, in Canada [26]. In a cross-sectional analysis of 775 healthy women from the Nurses’ Health Study, the aHEI was inversely associated with leptin and insulin concentrations but showed no association with other cardiometabolic risk factors traditionally associated with MetS [27]. These associations were, however, no longer present after adjusting for body mass index (BMI). Similar to the HEI-2015, this inverse association between the aHEI and the MetS is unlikely to be explained by variations in dietary SFA intake per se.

### 2.3. The Programme National Nutrition Santé Guideline Score (PNNSG-GS2) and MetS

The PNNS-GS2 is a score that reflects adherence to the 2017 French nutritional guidelines (Table 1) [28]. Briefly, it includes 13 components, of which seven are considered as adequacy recommendations and six refer to moderation recommendations. Similar to the aHEI, the PNNS-GS2 has no specific sub-score for SFA, but SFA intake is captured by the recommendation on added fat (to favor vegetable sources of fat vs. animal sources of fat) and indirectly by the recommendation on processed meats. Hence, individuals with a higher PNNS-GS2 consumed less SFA than individuals with a lower score [28]. Cross-sectional data from the Nutrinet-Santé Study have shown that a higher PNNS-GS2 was associated with a lower BMI, lower TG and glucose concentrations, lower systolic and diastolic blood pressures, and with higher concentrations of HDL-C in both men and women [28]. Recent data also suggest that higher adherence to the French dietary guidelines is prospectively associated with a lower risk of being overweight or obese [29]. These observations suggest that the PNNS-GS2 is likely to be inversely associated with the risk of incident MetS, but this needs to be formally confirmed with prospective data. The extent to which each of the components of the PNNS-GS2 are associated with individual features of the MetS is also unknown.

### 2.4. The Dietary Approaches to Stop Hypertension (DASH) Score and MetS

The Dietary Approaches to Stop Hypertension (DASH) score was created to measure adherence to the DASH diet, a healthy eating pattern that has been repeatedly associated with lower blood pressure and reduced CVD risk [30,31,32,33,34,35]. It was developed in the 1990s for the DASH trial, a randomized controlled trial in which more than 400 participants were randomized to either the control “American” diet, an “American” diet rich in fruits and vegetables or a DASH diet [36]. Most studies available at the time of the trial had shown no clear association between dietary fat (including SFA) and blood pressure [37]. However, it was argued that strict vegetarian diets (i.e., excluding dairy products) that were low in fat and SFA were associated with lower blood pressure. The DASH eating plan as we know it today promotes the consumption of fruits and vegetables, low-fat dairy products, whole grains, legumes, fish, poultry, and nuts and recommends limited intakes of sweets, SSBs, and red meats. Furthermore, individuals wanting to follow a DASH eating plan should choose foods that are low in sodium, SFA and trans-fat and rich in potassium, calcium, magnesium, fiber, and protein [34]. To that extent, the typical DASH diet is also a healthy eating pattern that is low in SFA [34].

Multiple versions of the DASH diet score are available in the literature [35,38,39,40]. Most scores include some of or all 8 food groups of the DASH eating plan, namely grains, meat/poultry/fish, vegetables, fruits, low-fat dairy products, fats and oils, nuts/legumes, and sweets. Additionally, most versions of the score include a component related to sodium intake. However, even if the recommendation to choose foods low in SFA and to limit consumption of SFA is included in the typical DASH eating plan, not all versions of the DASH score include a component directly related to SFA intake. For example, the DASH score created by Fung et al. does not include a component pertaining to SFA consumption based on the argument that it is already captured, at least partly, by the inclusion of red and processed meat components of the score [35].

In general, a greater adherence to a DASH score is associated with a lower prevalence of MetS [41,42]. Similar to the aHEI, the DASH score showed variable associations with the MetS and its key features among diverse Hispanic/Latino populations [43]. In a cross-sectional analysis among US women, higher adherence to the DASH eating plan was associated with lower TG concentrations, independent of BMI [27]. In a meta-analysis of available RCTs, consumption of the DASH diet over periods ranging from 2 to 24 weeks reduced systolic and diastolic blood pressure as well as LDL-C concentrations, but had no significant effect on TG, HDL-C, and glucose concentrations [44]. Interestingly, the reduction in blood pressure with the DASH diet has been shown to be similar among individuals with and without MetS [45]. Thus, when applied rigorously, the DASH diet is likely to have an impact on MetS, primarily through its important blood pressure lowering effect. Considering that the DASH diet is low in SFA by definition, it is unsurprising to find that adherence to this healthy dietary pattern also reduces serum LDL-C concentration, but this cannot explain the association between the DASH score and MetS.

### 2.5. The Mediterranean Diet and MetS

The concept of the MedDiet emerged from the Seven Countries Study in the 1950s, which unraveled particularly low CVD mortality rates in countries around the Mediterranean Sea [46]. Such low CVD rates were attributed, at least partly, to the intrinsically low SFA content of the diet of inhabitants around the Mediterranean (<7% E) compared with inhabitants from northern countries such as Finland [47]. It is now recognized that a MedDiet such as the one recommended in dietary guidelines is not just about SFA, although constitutively, a MedDiet is low in SFA. Typically, a MedDiet is characterized by very high intakes of plant-based foods, such as fruits, vegetables, cereals, beans, nuts and seeds, low to moderate intakes of fish and poultry and occasional consumption of eggs and red meat [48]. Olive oil is often considered the main source of fat in the MedDiet. A low to moderate wine consumption is also included in the MedDiet pattern, mostly consumed with meals [48]. The MedDiet is the most documented and researched healthy eating pattern. It has been repeatedly and consistently associated with lower risks of CVD, type 2 diabetes, and some types of cancer as well as cognitive-related diseases [49,50,51,52].

Several MedDiet scores have been created to measure adherence to a MedDiet. According to D’Alessandro and De Pergola, there is a certain degree of variability in the different MedDiet scores currently existing [53]. However, Galbete et al. found little differences in disease risk associations when comparing different MedDiet scores [54]. This is not entirely surprising considering that several core foods (fruit, vegetables, legumes, cereals, meat, dairy, fish, alcohol, and healthy fats) are found in most MedDiet scores [53,54]. It must also be stressed that the MedDiet emphasizes consumption of whole foods, with little if no focus on nutrients. Even if SFA is not part of most of the MedDiet scores per se, this healthy eating pattern is intrinsically low in SFA, in part due to the fact that this pattern focuses on fresh foods and because the recommendation to consume almost exclusively olive oil as a source of fat restricts in and of itself SFA consumption.

The PREDIMED study has shown in subjects without MetS at baseline that consumption of a MedDiet supplemented with either nuts or olive oil for five years had no impact on MetS incidence compared with a control diet. However, reversion occurred in almost 3 out of 10 of participants who had MetS at baseline in both groups consuming the MedDiet [55]. MedDiet participants supplemented with olive oil showed significant reductions in abdominal obesity and in fasting glucose while MedDiet participants supplemented with nuts showed a significant reduction in abdominal obesity only. Interestingly, increases in the biomarkers of foods supplied to the Mediterranean diet groups, namely oleic and α-linolenic acids, have been associated with the incidence, reversion, and prevalence of MetS [56]. Similar to the DASH eating plan, higher adherence to the MedDiet has been associated with lower TG concentrations among US women, independent of BMI [27]. Consumption of the MedDiet has also been associated with improvements in several features of the MetS, including blood lipids, blood pressure, glucose-insulin homeostasis, endothelial function, and inflammation markers [57]. Being nutrient-dense and having a low energy density, the MedDiet has often led to weight loss in intervention studies [58], thus potentially amplifying the cardiometabolic changes seen with the MedDiet [59]. Of note, the weight loss achieved with the MedDiet may be more important than with low-fat diets, but similar to low-carbohydrate diets [60]. Others have suggested that consumption of a MedDiet may also reduce central obesity, which in turn may contribute to reduced obesity-related and MetS-related disease risk [61]. To that extent, the contribution of the low SFA content of the MedDiet to its cardiometabolic benefits may be limited to LDL-C and not to features of the MetS.

## 3. Conclusions

Deciphering the impact of individual foods and nutrients on cardiometabolic risk factors such as those associated with MetS is very challenging because of the numerous food–nutrient interactions found within complex food patterns. This is certainly the case with SFA. In this short narrative review, we have shown that diet quality indices reflecting adherence to dietary guidelines (HEIs) or healthy dietary patterns (DASH, MedDiet) are quite consistently associated with a reduced risk of MetS. Data reviewed here provide indirect evidence that SFA in and of itself may play a rather limited role in the development of MetS. Indeed, while increased SFA intake in place of PUFA is unarguably associated with raised LDL-C concentrations, high LDL-C is not a typical feature of MetS. Moreover, the extent to which variations in SFA intake contribute to the cardiometabolic benefits associated with healthy eating patterns is likely to be diluted within the effects of numerous other nutrients and foods that constitute these patterns and which have been quite consistently associated with features of MetS. However, this may be of little concern since healthy eating scores that do not account for SFA intake per se inevitably capture this component through other components of the scores.

## Figures and Tables

**Table 1:** Components of different healthy eating indices.

HEI-2015	aHEI	PNNS-GS2
*Adequacy*		*Adequacy*
1. Total fruits	1. Fruits	1. Vegetables/Fruits
2. Whole fruits		
3. Total vegetables	2. Vegetables	
4. Greens and Beans	3. Nuts/Legumes	2. Nuts
		3. Legumes
5. Whole grains	4. Whole grains	4. Whole grain foods
6. Dairy products		5. Milk and dairy products
7. Total protein foods		
8. Seafood and Plant protein		6. Fish and sea foods
9. Fatty acids (UFA/SFA ratio)	5. Trans fat	7. Added fat (prefer vegetable sources)
	6. Long chain n3 fatty acids	
	7. PUFA	
*Moderation*		*Moderation*
	8. Red/Processed meat	8. Red meat
		9. Processed meat
10. Refined grains		
11. Sodium	9. Sodium	10. Sodium
12. Added sugars	10. SSBs	11. Sweet tasting beverages
		12. Sugary foods
13. SFA		
	11. Alcohol	13. Alcohol

HEI: Healthy Eating Index, PNNS-GS2: Programme National Nutrition Santé-Guideline Score updated to reflect the 2017 French dietary guidelines, UFA: Unsaturated fatty acids, SFA: Saturated fatty acids, PUFA: Polyunsaturated fatty acids, SSBs: Sugar-sweetened beverages. Note: The aHEI does not use the adequacy/moderation classification of its components.
